# Mental health literacy and help-seeking behaviour among Egyptian undergraduates: a cross-sectional national study

**DOI:** 10.1186/s12888-024-05620-7

**Published:** 2024-03-12

**Authors:** Mohamed Baklola, Mohamed Terra, Anhar Taha, Mohammed Elnemr, Mohammad Yaseen, Ahmed Maher, Ali Hussain Buzaid, Rahaf Alenazi, Safa Adil Osman Mohamed, Doaa Abdelhady, Abdel-Hady El-Gilany

**Affiliations:** 1 Public Health and Community Medicine Department, Faculty of Medicine, Mansoura University, Mansoura, Egypt; 2 Faculty of Medicine, Mansoura University, Mansoura, Egypt; 3 Faculty of Medicine, Alexandria University, Alexandria, Egypt; 4 Faculty of Medicine, Benha University, Benha, Egypt; 5 Faculty of Medicine, King Faisal University, Al-Hofuf, Saudi Arabia; 6 Medical intern, Buraydah central hospital, Al Qassim, Saudi Arabia; 7 Faculty of Medicine, National University, Khartoum, Sudan

**Keywords:** Mental Health literacy, Help-seeking behavior, Egyptian undergraduates, Mental well-being, Cross-sectional study, Gender disparities, Urban-rural differences

## Abstract

**Background:**

Mental health literacy (MHL) and help-seeking behaviors are pivotal in managing mental well-being, especially among Egyptian undergraduates. Despite the importance and prevalent psychological distress in this group, limited research has addressed MHL and associated behaviors in Egypt. This study aimed to assess the levels of MHL and help-seeking behavior among Egyptian university students.

**Methods:**

A cross-sectional study was conducted across ten Egyptian universities during the academic year 2022–2023. A convenience sample of 1740 students was obtained through online questionnaires distributed via social media platforms. The survey comprised demographic characteristics, the Mental Health Literacy Scale (MHLS), and the General Help Seeking Behavior Questionnaire (GHSPQ).

**Results:**

Among 1740 Egyptian undergraduates, medical students scored higher in recognizing disorders (*p* < 0.05), while non-medical students excelled in attitudes (*p* < 0.05). A strong correlation was observed between attitudes toward mental illness and total mental health literacy (coefficients of 0.664 and 0.657). Univariate analysis indicated a significant association with professional help-seeking (OR = 1.023). Females, individuals aged 21 or above, and non-medical students were more likely to seek mental health information (OR = 1.42, 1.82, 1.55 respectively). Help-seeking behavior for emotional problems was more inclined towards intimate partners, whereas suicidal thoughts prompted seeking professional help.

**Conclusion:**

The findings advocate for comprehensive mental health education, particularly in rural areas, and emphasis on the role of personal relationships in mental well-being. Implementing these insights could foster improved mental health outcomes and reduce related stigma in Egypt.

## Background

Mental health, a fundamental component of overall well-being, is characterized by an individual’s capacity to manage daily life stresses, contribute productively to work-related tasks, and actively participate within their community [1]. A related concept, mental health literacy (MHL), pertains to an individual’s knowledge and perceptions of mental health disorders. This entails the ability to identify disorders, manage symptoms, and initiate preventative measures [2]. Over time, the conceptualization of MHL has evolved to include an understanding of associated stigma [3].

Evaluating mental health literacy (MHL) in any population is a vital step in crafting a comprehensive knowledge map of mental health [3]. By evaluating the ability to recognize, manage, and prevent mental disorders, as well as understanding the stigma associated with these conditions, we gather invaluable insights into how society views and understands mental health issues [4]. This knowledge map plays a pivotal role in detailing the prevailing state of mental health awareness and can provide a basis for formulating targeted educational and interventional programs [5].

University students represent a particularly pertinent demographic for studying MHL. As young adults transitioning into greater responsibilities and independence, they often face unique stressors that can escalate mental health issues [6]. Moreover, their stage of life represents a period of significant intellectual growth and development of life-long attitudes and behaviors, including those related to mental health [6, 7]. Consequently, by directing attention toward this demographic, their MHL can be enhanced at a pivotal stage, with the potential for positive long-term influences on their mental health outcomes.

Recent evidence suggests a substantial prevalence of psychological distress, with 68.1% of university students in Egypt experiencing it. Additionally, there are significant barriers to mental health services utilization among these students [8]. Despite the high prevalence of these issues, mental health services are drastically lacking, particularly in low- and middle-income countries where services are estimated to be 200 times less prevalent than in high-income countries [9, 10]. In Egyptian universities, an alarming 37% of undergraduate students meet the diagnostic criteria for moderate depression, suggesting the significant impact of life-stage and academic stressors in this population [11].

Global studies have quantitatively evaluated MHL levels among university students. In the UK, the average score was 122.88 on the Mental Health Literacy Scale (MHLS) [12], while in Malaysia, Yogyakarta University students had a relatively poor mean score of 113.25 ± 12.22 [13]. In the Arab region, studies employing different assessment tools have been conducted. For example, a Saudi Arabian study at Jazan University found 90.3% of students exhibited intermediate MHL [14]. An earlier study in Iraq using the same tool found similar results [15]. The consequences of inadequate MHL are considerable, with improved MHL linked to better knowledge about help-seeking and reduced stigma towards mental illness [16]. Still, many students struggle to identify mental health symptoms, and 42.3% lack knowledge about available resources [12]. This lack of MHL can hinder students from recognizing their mental health issues and seeking suitable treatment, emphasizing the need for targeted interventions and further research.

In our study, ‘help-seeking behavior’ in the context of mental health is defined as the act of seeking assistance for mental health issues, which may involve accessing professional mental health services, consulting friends or family members, or engaging with support groups. This behavior is a pivotal component in effectively managing mental health problems and can substantially improve outcomes and overall quality of life [16]. However, levels of help-seeking behavior related to mental health remain alarmingly low. A study from Norway revealed that only 34% of adolescents displaying significant symptoms of depression and anxiety sought professional help in the preceding year [17]. According to a study conducted in Egypt, 90.3% of university students required professional mental health care, with 71.3% of them not seeking any form of help. Among those who sought assistance, 13.1% opted for religious methods, 11.2% sought professional mental health care, and 4.3% pursued alternative methods [8]. This disparity highlights the critical need for enhanced mental health literacy and accessible support systems to encourage more proactive and effective help-seeking behaviors.

The reluctance to seek professional help for common mental disorders has been attributed to a myriad of factors. These include negative attitudes towards help-seeking, logistical and cost concerns, societal barriers such as stigma, fear of judgement, loss of confidentiality, trivializing one’s symptoms, and skepticism about treatment efficacy [8, 18].

While global efforts have been made to assess Mental Health Literacy (MHL), a significant gap exists in MHL studies within Egypt, particularly regarding help-seeking behaviors among university students. Therefore, the objective of this study is to assess the levels of MHL and help-seeking behavior among Egyptian university students using standardized tools.

## Methods

### Study design and study period

A descriptive, cross-sectional study with an analytic component was conducted throughout the academic year 2022–2023 at ten Egyptian universities. The universities included are Cairo University, Mansoura University, Tanta University, Alexandria University, Assiut University, Al-Azhar University, Banha University, October 6 University, Ain Shams University, and the Misr University for Science and Technology. These institutions are situated in four distinct regions of Egypt, encompassing a broad spectrum of cultural and social milieus.

### Sample size

The study’s sample size was determined using Medcalc 15.8, considering the primary outcome of interest, which is the mean score on the Mental Health Literacy Scale (MHLS). A previous study in Saudi Arabia found an MHL score of 112.53 and SD = 12.64 [19]. With an alpha error of 0.01 and study power of 90%, then the sample size is 157 students. This is multiplied by a design effect of 10, then the final sample size is 1570 at least. The questionnaire was completed by 1740 participants.

### Sampling and data collection approach

The study utilized a convenience sampling approach after determining the required sample size at each selected university using the proportionate allocation technique. Data collection commenced on March 1, 2023, and continued until the sample was complete, utilizing Google forms for questionnaire administration. The survey was distributed to all students through official groups on various social media platforms, including the Telegram app, allowing participants to respond anonymously and at their own convenience. Additionally, one of the authors took on the responsibility of data collection at each university, posting the questionnaire in official groups across all academic years, ensuring coverage and reaching all students without imposing any obligation to participate. Participants were free to respond on their own time and maintain anonymity throughout the process.

### Study tools

The survey questionnaire comprised three sections, with the first section being the Demographic Characteristics e.g., sex, age, marital status, residence, living arrangement (whether away from family or not), family history of mental illness, and the educational levels of their father and mother. The second section included the Arabic version of the Mental Health Literacy Scale (MHLS), which has been adapted to suit the cultural and linguistic context of the Egyptian population. This scale comprises four factors: Attitude toward mental illness (Factor 1) with a reliability coefficient of 0.85, Attitude to someone with mental illness (Factor 2) with a reliability of 0.89, Recognition of disorders (Factor 3) at 0.79, and Mental illness information-seeking (Factor 4) with a reliability of 0.72 [19]. These reliability coefficients indicate a range from acceptable to excellent, demonstrating the scale’s robustness in measuring various aspects of mental health literacy. The Arabic version scale consists of 28 items for measurement of mental health literacy, it evaluates problems of recognition, awareness of help seeking behavior, awareness of reasons of mental health problems and their risk factors, awareness of self-treatments and the available professional treatments that health systems can provide, knowledge of how to promote good mental health and help seeking behaviors. MHLS has a good consistency with a test-retest reliability of 0.797 (*p* < 0.001) [19].

The development of the Arabic version of MHLS involved rigorous processes to ensure content and scale validity, alongside sufficient factor loadings, which are essential for the accurate assessment of mental health literacy. The adaptation process was guided by the principles outlined by Jorm et al. [20]., who recommend the vignette-interview design as the most common and effective approach to evaluate mental health literacy. This methodology ensures that the scale is not only linguistically but also contextually relevant to the target population, capturing the nuances and specificities of mental health perceptions and knowledge within the Egyptian context. Through this comprehensive adaptation and validation process, the Arabic version of MHLS used in our study is well-equipped to accurately assess the mental health literacy among Egyptian university students, ensuring that the findings are relevant, reliable, and reflective of the actual understanding and attitudes toward mental health within this demographic.

Lastly, the General help seeking behavior questionnaire (GHSPQ): To assess how much that degree of awareness affects their help seeking behavior [21]. The questions in this scale will be used to evaluate the intentions to seek help for mental health problems. GHSPQ has a good test-retest reliability (*r* = 0.92) [21].

### Statistical analysis

The data obtained from the survey were analyzed using the Statistical Package for Social Science Program (SPSS 25 for Windows). Descriptive statistical measures, including percentages, frequencies, means, and standard deviations, were used to summarize the demographic characteristics of the participants and describe each of the mental health literacy items. To examine the association between demographic variables and mental health literacy scores, as well as between medical and non-medical students, the Pearson chi-square test was employed. Moreover, Pearson correlation analysis was used to study the relationships between different factors of mental health literacy and help-seeking behavior. Binary logistic regression was applied to model the relationship between professional help-seeking behavior intention and mental health literacy factors. Additionally, the relationship between obtaining information related to mental illness and various sociodemographic variables was also modelled using binary logistic regression. The adjusted odds ratio, a measure used to compare the odds of an outcome between two groups, was calculated for each variable to assess its association with the listed factors. When the p-value is less than 0.05, the result is reported as statistically significant.

## Results

### Participant demographics

A total of 1740 undergraduate students from ten Egyptian universities participated in the survey, with an average age of 21.3 years (SD = 2.08) and a range of nine years. The majority were female (71.6%, *n* = 1,245) and medical students (57.6%, *n* = 1,002), with 42.4% (*n* = 738) being non-medical students. Most were single (88.7%, *n* = 1,544), and about 42.1% of mothers and 35.3% of fathers had secondary level education or less. A small fraction of participants (11.4%) indicated they would seek professional mental health care, while 16.9% preferred religious practices for managing mental health (Refer to Table 1 for details).

**Table 1:** Frequency distribution of demographic factors

Variables		Total Sample (n) column %	MHL Mean (SD)	p-value
**Sex**	Male	495 (28.4%)	91.3 (11.1)	< 0.05
	Female	1245 (71.6%)	95.3 (10.7)	
**Age Groups**	17–20	709 (40.7%)	94.3 (11.1)	< 0.05
	21 or above	1031 (59.3%)	94.1 (10.9)	
**Faculty**	Medical	1002 (57.6%)	94.9 (10.8)	0.161
	Non-medical	738 (42.4%)	93.2 (11.1)	
**Residence**	Urban	923 (53%)	94.9 (11.1)	< 0.05
	Rural	817 (47%)	93.4 (10.7)	
**Marital status**	Single	1544 (88.7%)	94.3 (11)	0.054
	Ever married	196 (11.3%)	93.1 (10.6)	
**Father’s education**	Secondary or below	615 (35.3%)	93.5 (10.8)	< 0.05
	Above secondary	1125 (64.7%)	94.6 (11)	
**Mother’s education**	Secondary or below	733 (42.1%)	93 (10.9)	< 0.05
	Above secondary	1007 (57.9%)	95 (10.9)	
**Methods of treatment used**	No	1171 (67.3%)	93.4 (10.4)	< 0.05
	Professional	199 (11.4%)	98.6 (12.5)	
	Religious	294 (16.9%)	93.2 (10.8)	
	Others	76 (4.4%)	98.2 (12.1)	
**Family history**	Yes	272 (15.6%)	96 (10.3)	< 0.05
	No	1468 (84.4%)	93.9 (11)	
**Highest Help-seeking intentions (E)** **Mean (SD)**		5.03 (1.84(		
**Highest Help-seeking intentions (S)** **Mean (SD)**		5.88 (2.15)		

### Mental health literacy scores (MHLS)

Table 2 provides a comparative analysis of mental health literacy scores between medical and non-medical students. In Factor 1, assessing attitudes toward mental illness, medical students scored significantly higher in items 14, 15, 19, 20, and 21 (*p* < 0.05), with no significant differences in items 16, 17, and 18. For Factor 2, non-medical students scored higher in items 22 to 25 and 27 (*p* < 0.05), with similar scores in item 26 and no discrepancy in item 28 (*p* = 0.897). Medical students significantly outperformed non-medical students in Factor 3, recognizing mental disorders across items 1 to 9 (*p* < 0.05). In Factor 4, concerning information-seeking behaviors, significant differences were noted in items 10 and 12, while items 11 and 13 showed no difference. Despite these variations, overall mental health literacy scores revealed no significant differences between the groups.

**Table 2:** Mental health literacy scale subsections among medical and non-medical students

Mental Health Literacy Indicators	Medical Mean (SD)	Non-medical Mean (SD)	p-value
**Factor 1: Attitude toward mental illness**			
Q14. Belief that mental illness signifies personal weakness.^*^	3.97 (1.200)	3.50 (1.372)	**< 0.05**
Q15. Perception of mental illness as not a real medical condition. ^*^	4.72 (0.798)	4.56 (0.955)	**< 0.05**
Q16. View of people with mental illness as dangerous. ^*^	3.51 (1.119)	3.58 (1.088)	0.160
Q17. Tendency to avoid people with mental illness. ^*^	4.27 (0.973)	4.21 (1.068)	0.219
Q18 Reluctance to disclose one’s own mental illness. ^*^	3.50 (1.227)	3.45 (1.253)	0.766
Q19. Belief that seeing a mental health professional indicates weakness. ^*^	4.42 (1.085)	4.10 (1.324)	**< 0.05**
Q20. Unwillingness to seek professional help for mental illness. ^*^	4.21 (1.118)	4.03 (1.182)	**< 0.05**
Q21. Skepticism about the effectiveness of professional mental health treatment. ^*^	4.26 (0.972)	3.92 (1.154)	**< 0.05**
**Factor 2: Attitude to someone with mental illness**			
Q22. How willing would you be to move next door to someone with a mental illness?	2.72 (0.995)	2.93 (1.037)	**< 0.05**
Q23. How willing would you be to spend an evening socializing with someone with a mental illness?	3.25 (1.126)	3.49 (1.104)	**< 0.05**
Q24. How willing would you be to make friends with someone with a mental illness?	3.21 (1.093)	3.43 (1.122)	**< 0.05**
Q25. How willing would you be to have someone with a mental illness start working closely with you on a job?	3.29 (1.033)	3.41 (1.084)	0.034
Q26. How willing would you be to have someone with a mental illness marry into your family?	2.29 (1.045)	2.49 (1.130)	**< 0.05**
Q27. How willing would you be to vote for a politician if you knew they had suffered a mental illness?	2.32 (1.191)	2.32 (1.282)	**< 0.05**
Q28. How willing would you be to employ someone if you knew they had a mental illness?	3.05 (1.051)	3.07 (1.078)	0.897
**Factor 3: Recognition of disorders**			
Q1. If someone became extremely nervous or anxious… to what extent do you think it is likely they have Social Phobia?	3.27 (0.712)	3.17 (0.830)	**< 0.05**
Q2. If someone experienced excessive worry… to what extent do you think it is likely they have Generalized Anxiety Disorder?	3.60 (0.684)	3.44 (0.739)	**< 0.05**
Q3. If someone experienced a low mood for two or more weeks… to what extent do you think it is likely they have Major Depressive Disorder?	3.14 (0.783)	2.99 (0.837)	**< 0.05**
Q4. To what extent do you think it is likely that Personality Disorders are a category of mental illness?	3.32 (0.791)	3.11 (0.868)	**< 0.05**
Q5. To what extent do you think it is likely that Dysthymia is a disorder?	3.18 (0.798)	3.05 (0.842)	**< 0.05**
Q6. To what extent do you think it is likely that the diagnosis of Agoraphobia…?	2.94 (0.896)	2.84 (0.975)	**< 0.05**
Q7. To what extent do you think it is likely that the diagnosis of bipolar disorder…?	3.13 (0.868)	2.94 (0.909)	**< 0.05**
Q8. To what extent do you think it is likely that Cognitive Behavior Therapy (CBT)…?	3.46 (0.747)	3.26 (0.838)	**< 0.05**
Q9. To what extent… mental health professional to break confidentiality: if you are at immediate risk of harm to yourself or others? ^*^	1.56 (0.867)	1.77 (1.003)	**< 0.05**
**Factor 4: Mental illness information-seeking**			
Q10. I am confident that I know where to seek information about in mental illness.	3.38 (1.133)	3.09 (1.111)	**< 0.05**
Q11. I am confident using the computer or telephone to seek information about mental illness.	3.64 (1.097)	3.64 (1.139)	0.316
Q12. I am confident attending face to face appointments to seek information about mental illness (e.g., seeing the GP).	3.54 (1.227)	3.67 (1.258)	**< 0.05**
Q13. I am confident I have access to resources (e.g., GP, internet, friends) that I can use to seek information about mental illness.	3.77 (1.098)	3.73 (1.111)	0.109
**Overall mental health literacy (combined score of all factors)**	93.18 (11.13)	94.93 (10.75)	0.16

*Reverse scored questions

### Correlation between mental health literacy and help-seeking behaviors

Table 3 demonstrates the Pearson correlation between mental health literacy factors and help-seeking behavior. There is a strong positive correlation between attitudes toward mental illness (a) and total mental health literacy (e), and attitudes toward individuals with mental illness (b) and total mental health literacy (e), with coefficients of 0.664 and 0.657. Positive correlations also exist between recognition of disorders (c), information-seeking (d), and total mental health literacy, with coefficients between 0.511 and 0.570. Weak correlations are noted between total mental health literacy and professional help-seeking for emotional problems (f), with a slight negative correlation regarding suicidal thoughts (g), but not significant. The correlations between different mental health literacy factors (a through d) are positive but weaker, ranging from 0.078 to 0.250. The table encapsulates a multifaceted relationship between mental health literacy factors and help-seeking behaviors, showing both positive and negative correlations with varying strengths.

**Table 3:** Pearson correlation between factors of mental health literacy and help-seeking behavior

Variables		a	b	c	d	e	f	g
**Factors of** **MHLS**	**Attitude toward mental illness (a)**	1	0.125^**^	0.149^**^	0.198^**^	0.664^**^	0.008	-0.007
	**Attitude to someone with mental illness (b)**	0.125^**^	1	0.078^**^	0.181^**^	0.657^**^	0.064^**^	0.015
	**Recognition of disorders (c)**	0.149^**^	0.078^**^	1	0.250^**^	0.511^**^	0.008	-0.014
	**Mental illness information-seeking (d)**	0.198^**^	0.181^**^	0.250^**^	1	0.570^**^	-0.002	-0.029
**Total mental health literacy (e)**		0.664^**^	0.657^**^	0.511^**^	0.570^**^	1	0.039	-0.009
**Highest Professional help-seeking intention (emotional) (f)**		0.008	0.064^**^	0.008	-0.002	0.039	1	0.592^**^
**Highest Professional help-seeking intention (suicidal) (g)**		-0.007	0.015	-0.014	-0.029	-0.009	0.592^**^	1

*p < 0.005, **p < 0.001

### Association of mental health literacy factors with professional help-seeking behavior intention

In the univariate regression analysis, Factor 2 (F2) displayed a significant association with professional help-seeking behavior intention, with an odds ratio (OR) of 1.023. However, Factors 1 (F1), 3 (F3), and 4 (F4) did not show a significant relationship with professional help-seeking behavior intention (Table 4).

**Table 4:** Binary logistic regression analysis of professional help-seeking behavior intention regarding mental health literacy factors

Variables	Professional help-seeking behavior intention*		
	Univariate Regression		
	OR 95%	C.I	P-Value
F1	1.008	(0.988–1.029)	0.43
F2	1.023	)1.003–1.043)	**< 0.05**
F3	1.022	) 0.991–1.055)	0.171
F4	0.996	(0.963–1.031)	0.819

*The dichotomous dependent variable (Yes, No)

### Sociodemographic factors as predictors of getting information related to mental illness

In both the univariate and multivariate regression analyses, several variables were found to be statistically significant. Females demonstrated a higher likelihood of seeking mental health information in both univariate (OR = 1.42) and multivariate analysis (AOR = 1.35). Similarly, individuals aged 21 or above showed a higher likelihood of seeking mental health information compared to those aged 17–20 in both univariate (OR = 1.82) and multivariate analysis (AOR = 1.76). Non-medical students displayed a higher likelihood of seeking mental health information in univariate analysis (OR = 1.55), but this was not significant in the multivariate analysis. Participants from rural residences and those ever married were more likely to seek mental health information in both univariate and multivariate analyses (*p* < 0.05). Additionally, mother’s education above secondary level and having a family history related to mental illness were associated with higher likelihoods of seeking mental health information in both univariate and multivariate analyses (*p* < 0.05). However, other variables such as professional and religious methods of treatment, and father’s education were not found to be significant in the multivariate regression analysis (Table 5).

**Table 5:** Binary logistic regression analysis of get information related to mental illness regarding sociodemographic

Variables		Get information related to mental illness*			
		Univariate Regression		Multivariate Regression	
		OR 95% (C.I)	P-Value	OR 95% (C.I)	P-Value
**Sex**	Male	1 (r)	**< 0.05**	1 (r)	**< 0.05**
	Female	1.42 (1.07–1.89)		1.35 (1-1.82)	
**Age groups**	17–20	1 (r)	**< 0.05**	1 (r)	**< 0.05**
	21 or above	1.82 (1.39–2.38)		1.76 (1.32–2.34)	
**Faculty**	Medical	1 (r)	**< 0.05**	1 (r)	0.693
	Non-medical	1.55 (1.18–2.03)		0.99 (0.94–1.04)	
**Residence**	Urban	1 (r)	**< 0.05**	1 (r)	**< 0.05**
	Rural	1.57 (1.2–2.06)		1.4 (1.05–1.86)	
**Marital status**	Single	1 (r)	**< 0.05**	1 (r)	**< 0.05**
	Ever married	2.15 (1.25–3.71)		1.79 (1.01–3.17)	
**Methods of treatment used**	No	1 (r)			
	Professional	0.55 (0.28–1.07)	0.08		
	Religious	1.25 (0.39–3.98)	0.69		
	Others	0.34 (0.19–0.61)	**< 0.05**	0.37 (0.2–0.68)	**< 0.05**
**Father’s education**	Secondary or below	1 (r)	**< 0.05**	1 (r)	0.146
	Above secondary	1.8 (1.37–2.36)		1.28 (0.91–1.8)	
**Mother’s education**	Secondary or below	1 (r)	**< 0.05**	1 (r)	**< 0.05**
	Above secondary	1.98 (1.51–2.6)		1.7 (1.21–2.4)	
**Is there any medical history in the family related to mental illness?**	No	1 (r)	**< 0.05**	1 (r)	**< 0.05**
	Yes	2.07 (1.31–3.28)		1.81 (1.13–2.9)	

*The dichotomous dependent variable (Yes, No)

### Help-seeking intentions for emotional problems and suicidal thoughts

**Fig. 1:**
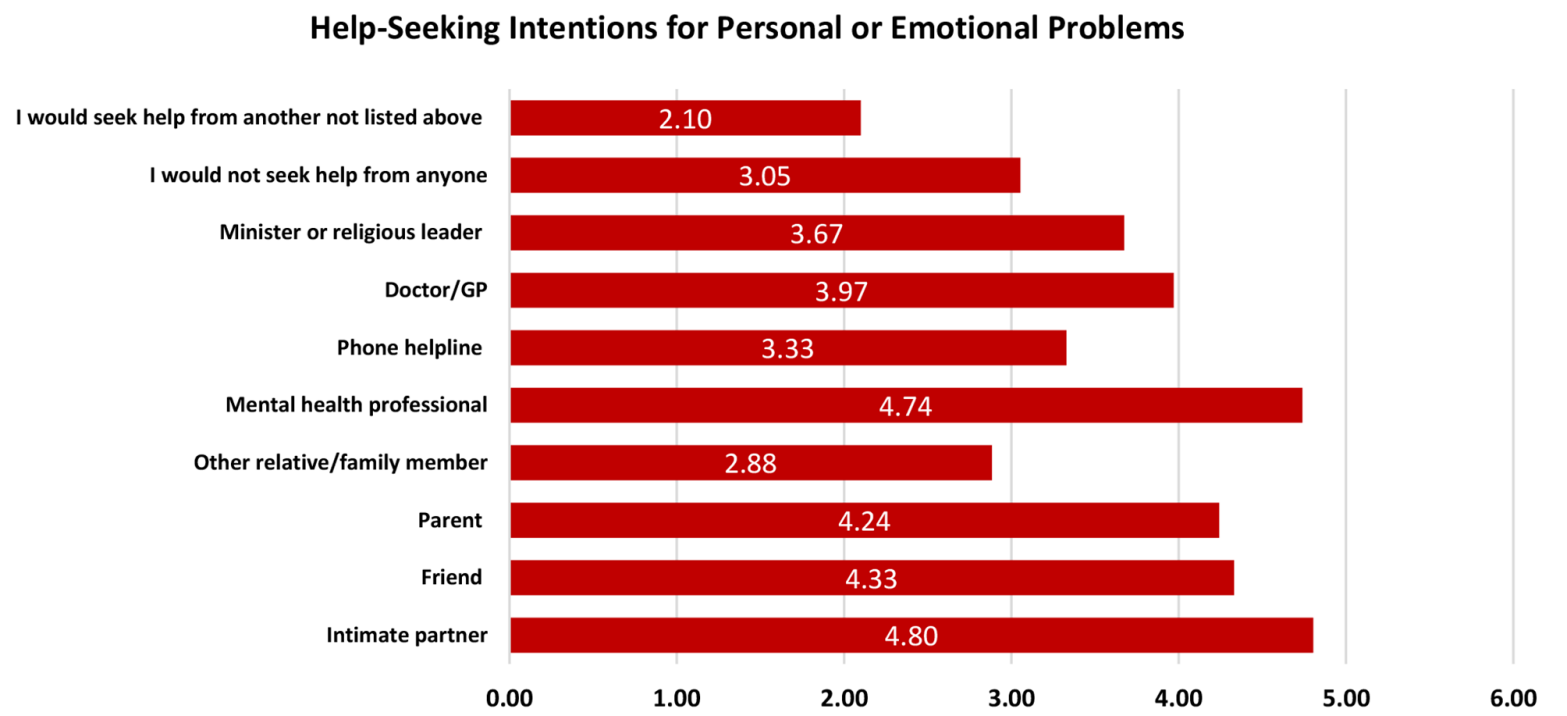
Help-seeking intentions for personal or emotional problems

Figure 1 shows participants’ help-seeking intentions for personal or emotional problems, highlighting intimate partners as the primary source of support (4.80), followed by mental health professionals (4.74), friends (4.33), and parents (4.24). Doctors/GPs and ministers or religious leaders are rated lower at 3.97 and 3.67, respectively, with other relatives and unlisted options being the least preferred (2.88 and 2.10). This indicates a preference for close personal connections and professional mental health resources.

**Fig. 2:**
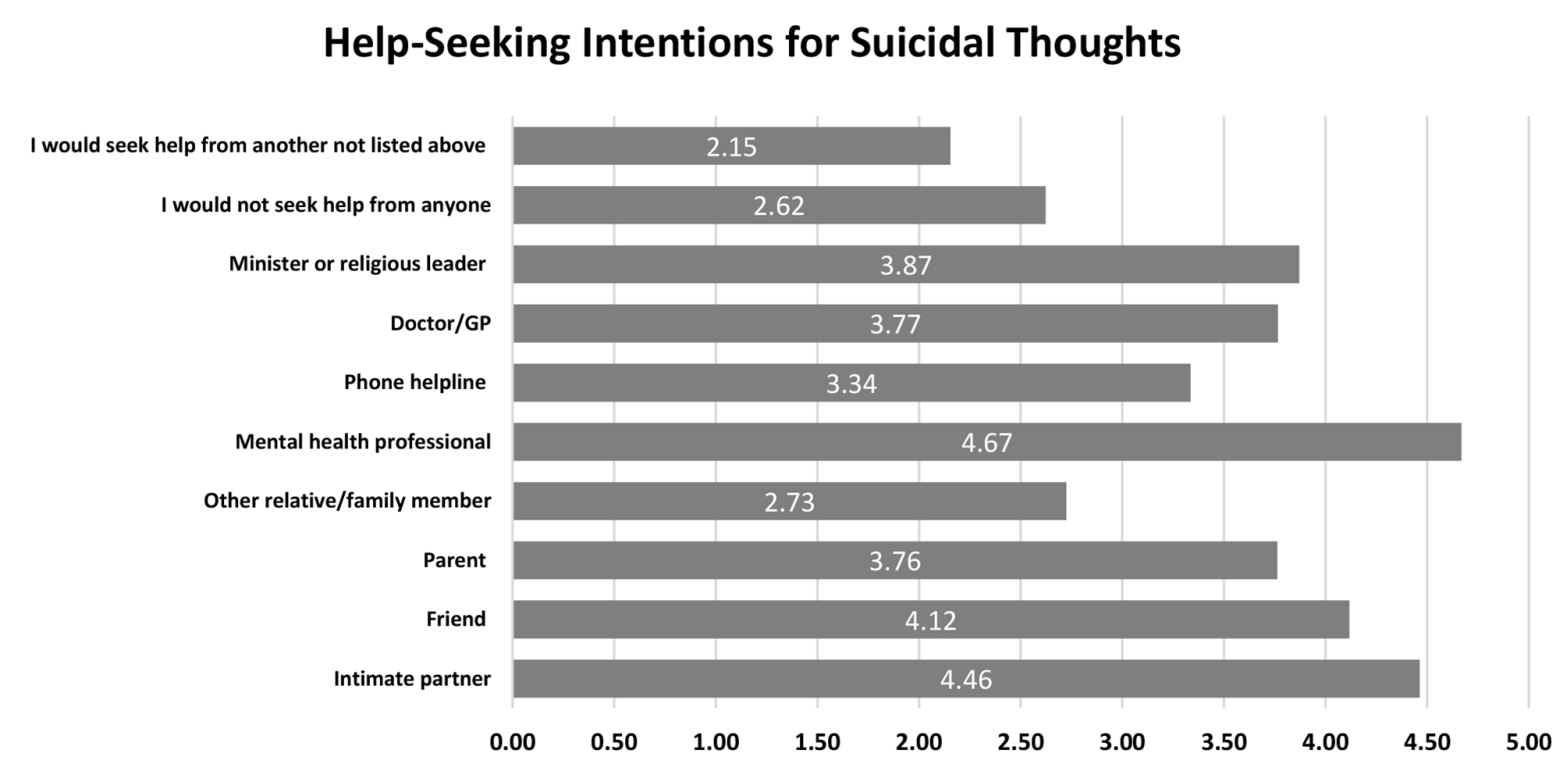
Help-seeking intentions for suicidal thoughts

Figure 2 outlines the help-seeking intentions for suicidal thoughts, emphasizing mental health professionals (4.67), intimate partners (4.46), and friends (4.14). Doctor/GP and parents are noted at 3.77 and 3.76, with ministers or religious leaders rated slightly higher at 3.87. Other relatives, phone helplines, and unlisted options have lower ratings (2.73, 3.34, and 2.15, respectively). This pattern underscores the value of professional help in severe mental health cases while maintaining the importance of personal support networks.

## Discussion

The present study sought to explore mental health literacy among Egyptian undergraduate students and determine its impact on their help-seeking behavior. It was the first study to explore mental health literacy among any sector of Egyptian population. The overall scores revealed intricate correlations between various mental health literacy factors, with coefficients ranging from 0.078 to 0.664, demonstrating the complex interplay between attitudes, recognition of disorders, and information-seeking behaviors.

The comparative analysis between medical and non-medical students, which exhibited no significant differences in overall mental health literacy scores, uncovers a common ground in understanding that can be exploited for future educational interventions. While our comparative analysis found no significant differences in overall mental health literacy between medical and non-medical students, this is a notable deviation from studies in other regions. For instance, a study in India reported higher literacy among medical students, highlighting the influence of curriculum differences and cultural factors in shaping mental health knowledge [22]. Moreover, the study emphasizes a concerning trend of preference for religious practices (16.9%) over professional mental health care (11.4%), reflecting cultural influences and potential barriers to professional help-seeking.

Sex emerged as an influential factor in mental health literacy, with female students exhibiting higher levels compared to their male counterparts. This observation is consistent with findings from a study in Saudia Arabia [19]. Our study reveals a significant difference in Mental Health Literacy (MHL) scores between urban and rural students, with urban students scoring higher. Furthermore, the disparity in literacy levels between urban and rural students echoes observations in a Brazilian study, underscoring the impact of socioeconomic factors and access to resources [23]. A similar pattern was observed by Griffiths et al. (2009) in regions with limited access to mental health awareness initiatives [24]. This highlights the role of residency in influencing mental health literacy in relation to available resources.

Parental education levels played a pivotal role in mental health literacy, with students having parents with higher education showing better mental health literacy scores. This emphasizes the significant role of parental education in influencing the mental development and well-being of their children [25].

A noteworthy difference was found among students who sought professional help, religious help, other treatments, or no help at all. A significant proportion did not seek any kind of treatment, while a smaller percentage pursued professional advice. Mental health literacy was higher among those who sought professional treatment compared to those who did not. This emphasizes the connection between mental health literacy and help-seeking behaviors. The correlation between mental health literacy and professional help-seeking behavior in our study aligns with findings from a study in Saudi Arabia and a Swiss study [19, 26] [19, 26]. On a group of 10 students a trial conducted to experience the effect of being aware of mental health problems and the influence of that awareness on this group ability to recognize mental health problems, and when to seek help, and the results were better mental health literacy, and good recognition of mental health problem that require help seeking from a professional [27].

Our observation that a family history of psychiatric disease enhances mental health literacy resonates with findings from a Danish study, suggesting that familial exposure to mental health issues fosters greater awareness and understanding [28]. This relationship might be explained by more open conversations about mental health within these families, or direct exposure to psychiatric care, thus demystifying the process. However, this increased literacy may not necessarily translate to a more positive attitude towards mental health, as stigma and negative perceptions could still exist. The connection between family history and mental health literacy underscores the complexity of mental health education, indicating that it is not merely a matter of information but is intertwined with personal experience and cultural context [29].

The present study investigated the differences in mental health literacy (MHLS) and help-seeking behavior among Egyptian medical and non-medical undergraduates. The analysis reveals nuanced insights into both the similarities and disparities between these two groups. Medical students scored an MHLS of 93.18, while non-medical students scored slightly higher at 94.93 with no statistically significant difference between the two groups. These findings may seem counterintuitive at first, as one might expect medical students to have a higher literacy rate due to their specialized education. However, the results suggest that non-medical students may access alternative sources of knowledge other than medical education. This observation is supported by prior studies, which found no significant difference in mental health literacy, and T reported no difference in the stigmatization of psychiatric diseases between the two groups [14, 30].

When delving into the factors affecting mental health literacy, such as attitudes towards mental illness, interaction with those with mental illness, information seeking, and recognition of disorders, we uncovered some key distinctions. Medical students generally scored higher on questions related to personal weaknesses, real medical illness, strength, and treatment effectiveness. They also had significantly higher mean scores for recognizing a wide range of mental health conditions and understanding therapeutic approaches such as Cognitive Behavior Therapy (CBT). These differences may reflect the specialized training and exposure that medical students receive, enhancing their understanding of these complex topics [31].

Interestingly, no significant differences were observed in beliefs about danger, avoidance, secrecy, or unwillingness to seek professional help. This could indicate common societal perceptions or cultural attitudes towards mental illness shared by both groups, regardless of their educational background.

Non-medical students exhibited a greater willingness to socialize with individuals with a mental illness, reflecting potentially more empathetic or open attitudes. This could be explored further to understand the underlying factors driving this difference, as it may offer important insights into stigma reduction strategies. The study also found variations in confidence levels between the two groups in information-seeking related to mental health. While medical students were more assured in identifying sources for mental illness information, non-medical students were more confident in face-to-face appointments. Both groups, however, were equally confident in utilizing technology for this purpose.

Our findings contribute to a broader understanding of mental health literacy and attitudes among university students. The nuanced differences between medical and non-medical students may have implications for targeted educational interventions and awareness campaigns. Tailoring strategies to each group’s specific needs and understandings can foster greater mental health literacy and reduce stigma, ultimately promoting a more informed and compassionate society.

Our study found a strong positive correlation between mental health literacy and attitudes towards mental illness, contrasting with previous findings [32, 33]. Our findings on attitudes towards mental illness and the recognition of disorders show similarities to a study in united states [34], yet differ from a study in Korea [35], highlighting the role of cultural and educational contexts in shaping these aspects of mental health literacy. The inconsistency may arise from differing methodologies in measurement. While we employed a survey with specific questions, the German study used interviews, and another used a specialized questionnaire. These differences in approach highlight the complexity in assessing attitudes towards mental health and emphasize the importance of consistent measurement techniques to compare across studies.

In our study, recognition of mental disorders was moderately correlated with mental health literacy, aligning with a Nigerian study, which reported low literacy among undergraduates, particularly in recognizing schizophrenia and differentiating it from depression [36]. However, this correlation contrasts with an Iranian study, which found no specific link between disease recognition and mental health literacy, illustrating potential cultural or methodological differences that may impact the understanding of these relationships [37].

A notable finding in our study was the moderate positive correlation between information seeking and overall mental health literacy. Supported by research on internet-provided information [38], this emphasizes the importance of accessible and accurate online information in promoting mental health literacy. Furthermore, we observed positive correlations between total mental health literacy and professional help-seeking intention, aligning with previous work [12]. Additionally, there was a strong positive correlation between help-seeking intentions for emotional problems and suicidal thoughts, a relationship that echoes prior research [39].

We utilized a binary logistic regression analysis to determine the significant predictors of professional help-seeking intention among undergraduates. Of the variables analyzed, the attitude towards someone with a mental illness was the most influential in determining the probability of professional help-seeking intention. Conversely, variables such as attitude towards mental illness, recognition of disorders, and information seeking did not significantly influence this intention. These findings concur with a previous systematic review and meta-analysis, which indicated an association between personal stigma and reduced help-seeking behaviour [40]. However, they contradict findings of a 3-years prospective cohort study that found no direct link between personal stigma and help-seeking behaviours [41].

Our study further investigated sociodemographic variables and their influence on information acquisition related to mental illness. Females demonstrated a significant propensity to seek mental health information, aligning with our results that suggest better mental health literacy in females compared to males. A positive correlation between mental health literacy and information seeking also emerged from the Pearson correlation table, corroborating our findings. We observed that students aged 21 years and above were more inclined to seek mental health information. A potential reason could be the distinctions in cognitive and emotional maturity, as declared in an old study conducted in by in Texas in 1980 which found that older undergraduates often exhibited traits of confidence, emotional and social adjustment, and analytical problem-solving, potentially contributing to the observed disparity in information-seeking behaviors [42]. The influence of sociodemographic factors on information-seeking behavior in our study aligns with findings from a study in Turkey [43], yet diverges from observations in a study in Nigeria [44], reflecting varying societal norms and information access.

Interestingly, while non-medical students showed a significant influence on information seeking in univariate analysis, this significance dissipated in multivariate analysis. Marital status also played a role; those ever married exhibited a greater inclination towards information seeking, possibly due to heightened feelings of responsibility, as suggested by an American study that discussed responsibilities related to parenthood conducted in 2011 [45].

Further, a family history of mental health problems was a significant predictor of information-seeking behavior. This is consistent with our observation that students with such histories possess higher mental health literacy than their counterparts without. Parental education was another significant influencer, with our findings resonating with a study conducted in united states which determined that students with more educated parents exhibited better mental health [46]. Lastly, students resorting to non-professional treatments were the least likely to seek information about mental health issues, potentially owing to misinformed beliefs about mental illness. This underscores the importance of improving mental health literacy and dispelling myths surrounding non-conventional treatments.

This study underscored the pivotal role of intimate partners in the support system for individuals dealing with personal or emotional problems. This could reflect a cultural aspect where personal and intimate relationships are highly valued and trusted for emotional support. The prominence of mental health professionals and friends in the ratings also highlights a growing acceptance and recognition of professional mental healthcare, along with the traditional reliance on peer support. Conversely, the lower ratings for Doctor/GP and phone helplines may indicate a perceived lack of specialization or accessibility in these channels for emotional or personal issues. Our findings on help-seeking intentions, particularly the reliance on intimate partners and mental health professionals, align with a study on the role of partner influence on Help-seeking for mental health concerns [47], yet differ from a study in Australian [48], illustrating cultural variations in preferred sources of support for mental health issues.

And also, emphasized that help seeking intentions shifts notably towards mental health professionals when dealing with suicidal thoughts, reflecting an understanding of the severity of such issues and the importance of specialized care. The continued significance of intimate partners in these ratings underscores their vital role as initial support, possibly due to immediate accessibility and deeply ingrained trust. The diminished reliance on other family members, doctors, and helplines in this context might highlight perceived inadequacies in these support systems for handling such a critical mental health crisis. Together, these findings suggest a nuanced understanding of mental health and help-seeking behavior in the sample population, recognizing the importance of both personal relationships and professional expertise. These insights could inform targeted interventions and awareness campaigns, emphasizing the roles that different support systems can play in various mental health scenarios. Furthermore, they may inspire improvements in mental health literacy, particularly emphasizing the vital roles that professional mental health services can play in supporting individuals facing severe mental health challenges. A previous Canadian study supports these two findings, they found that students intend to seek help from their friends for their emotional problems and higher intentions to seek help from professional health care if they had suicidal thoughts [39].

### Limitations

The study faced several limitations that should be considered when interpreting the findings. The use of a convenience sampling approach, while efficient, might have attracted participants particularly interested in mental health, thereby limiting the generalizability of the results to all Egyptian undergraduates. This approach could also lead to an overrepresentation of certain universities or academic disciplines. The reliance on self-reported measures, including the Mental Health Literacy Scale (MHLS) and General Help Seeking Behavior Questionnaire (GHSPQ), may have been influenced by social desirability bias, potentially skewing the accuracy of responses. The cross-sectional design of the study poses constraints on establishing causal relationships between mental health literacy and help-seeking behavior. Additionally, the focus on university students may restrict the applicability of the findings to other demographic groups within Egypt, including those not pursuing higher education. Despite online distribution, the reach might still have been constrained to students with internet access, possibly omitting some segments of the population. Nevertheless, the study offers a significant contribution to understanding mental health literacy and help-seeking tendencies among Egyptian undergraduates, and the findings provide a solid foundation for further research and the development of targeted interventions.

## Conclusion

This comprehensive study on mental health literacy (MHL) and help-seeking behavior among Egyptian undergraduates highlights important gender, urban-rural, and educational disparities in MHL. The absence of differences between medical and non-medical students points to a broader scope of mental health education. The positive correlation between MHL and professional help-seeking emphasizes the need for targeted educational interventions. Personal relationships also play a vital role in seeking help for mental health issues. These findings have significant implications for the development of mental health promotion strategies in Egypt, including the need for rural-focused awareness campaigns and open conversations within intimate relationships. While insightful, the results should be considered with awareness of limitations related to sampling and self-reporting. Overall, the study forms a vital step towards a more informed and resilient approach to mental well-being in Egypt.

## Data Availability

The datasets used during the current study are available from the corresponding author upon reasonable request.
